# Fire

**Published:** 1871-07

**Authors:** 


### The Bistoury.

ELMIRA, N. Y., JULY, 1871.

The BISTOURY is published Quarterly, upon the 1st of April, July, October and January, at Fifty Cents a year, in advance.

### FIRE.

Since the last issue of the BISTOURY we have met with a great misfortune, in the loss of our Eye and Ear Institute, by fire.

Our fine collection of surgical instruments, large medical library, cabinet of pathological specimens and skeletons, manikins, models, paintings, and everything that contributed so much to our pleasure and convenience were entirely consumed. Many objects of interest and usefulness can never be replaced—they were the accumulation of years of active practice in our profession. Our notes of rare cases of disease, personal formulæ, history of surgical operations performed, specimens of malignant growths, tumors, &c., are losses that cannot be replaced with money.

The confusion in our businesss consequent upon being compelled to seek other and more contracted quarters, with no private sanctum in which to pen our usual communications to our readers, must be a sufficient apology both for the lateness of the issue and the meagre amount of editorial matter.

By the next issue of the BISTOURY we will occupy our new and elegant quarters, at the old place, when we will be prepared to welcome our friends to our new editorial apartments, where we hope to be able to write for our magazine with pleasure to ourself and profit to our readers.

